# The relationship between self-efficacy/resilience and the mental health status of hemodialysis nurses: multiple mediation effects of insomnia and fatigue

**DOI:** 10.3389/fpsyg.2025.1618422

**Published:** 2025-09-24

**Authors:** Wen-Xuan Zhang, Xiao-Qing Ye, Sui-Qin Wen, Rao-Ping Wang, Ying-Hua Li

**Affiliations:** ^1^Department of Maternal and Child Health, School of Public Health, Tongji Medical College, Huazhong University of Science and Technology, Wuhan, China; ^2^Department of Epidemiology, School of Public Health, Sun Yat-sen University, Guangzhou, China; ^3^Nephrology Center, The Seventh Affiliated Hospital of Sun Yat-sen University, Shenzhen, China; ^4^Department of Nephrology, The Blood Purification Center, The First Affiliated Hospital of Sun Yat-sen University, Guangzhou, China

**Keywords:** self-efficacy, resilience, mental health status, hemodialysis nurses, multiple mediation effects, structural equation model (SEM)

## Abstract

**Background and purpose:**

Due to the high frequency of hemodialysis treatment, hemodialysis nurses have to bear a lot of psychological pressure, which makes them more vulnerable to anxiety and depression. This study aims to explore the relationship between self-efficacy/resilience and the mental health status of hemodialysis nurses and mediating roles of insomnia and fatigue.

**Methods:**

A cross-sectional study was conducted in hemodialysis rooms/centers in Guangdong Province, China, including 1630 nurses aged between 18 and 59. Structural equation model (SEM) was used to explore the relationship between self-efficacy/resilience and the mental health status of hemodialysis nurses, as well as multiple mediation effects of insomnia and fatigue.

**Results:**

The results of multiple mediation effects analysis based on SEM suggested that the above-mentioned factors can directly affect the mental health status of hemodialysis nurses. Furthermore, insomnia and fatigue played multiple mediating roles in the pathway of self-efficacy/resilience and the mental health status of hemodialysis nurses.

**Conclusion:**

The specific demands of hemodialysis nursing bring various stressors that can negatively affect the mental health status of hemodialysis nurses, which makes them vulnerable to negative emotions. Therefore, finding ways to improve self-efficacy and resilience, and take care of sleep quality to reduce fatigue is essential to improve the mental health status of hemodialysis nurses.

## 1 Introduction

Chronic kidney disease (CKD) will be the fifth leading cause of death globally by 2040 (Foreman et al., 2018; Li et al., 2021). At present, the global prevalence of CKD is 8%∼16%, making it a threatening condition for public health and consuming many medical resources (Floege and Johnson, 2021). According to the data from the national epidemiological survey, the number of CKD patients in China is around 119.5 million, and the prevalence is 10.8% (Liu, 2013). With the progression of the disease, CKD will eventually develop into End-Stage Renal Disease (ESRD), which is a condition that also poses many challenges to patients and national health systems.

According to a survey in China, there are about 2 million ESRD patients in the country, with an average annual growth rate of 10%∼12% in the next 10 years (Shen et al., 2017). Current treatments for ESRD mainly include hemodialysis and peritoneal dialysis, accounting for 70%∼80% of all patients with renal replacement therapy (Wark, 2020). Due to the long duration and high frequency of hemodialysis treatment (Watanabe et al., 2021), hemodialysis nurses need to work more efficiently and invest more energy in their daily routine. This means that nurses have to bear a lot of psychological pressure, which makes them more vulnerable to anxiety and depression (Sriharan et al., 2021). In addition, nurses often need to stick to their posts as a result of patients’ sudden illness, which can undoubtedly increase their overtime hours. According to Walton et al. (2020), these pressuring factors in the work environment lead to anxiety, depression, post-traumatic stress disorder (PTSD), and other psychological problems that may even include suicidal tendencies. Especially during the COVID-19 pandemic, they have to face the risk of infection and fear of death (Isha et al., 2023; Yıldırım and Şentürk, 2023). These affect not only the mental health of nurses but the efficiency of care for patients, leaving them prone to cause serious medical errors (Saleem et al., 2022).

Self-efficacy refers to an individual’s perception and belief about their abilities to complete specific tasks (Abusubhiah et al., 2023). Individuals with higher self-efficacy are more able to effectively perceive and recognize the emotions of others and regulate their own negative emotions, which makes them less likely to develop anxiety and depression (Wang et al., 2019). A longitudinal study (Maciejewski et al., 2000) conducted in the United States found that self-efficacy has a negatively predictive effect on depression, and people with high self-efficacy have a lower degree of depressive symptoms. Other related studies also found that self-efficacy can negatively predict insomnia and fatigue. A study designed by Byun et al. (2020) suggested that self-efficacy is an influencing factor of insomnia and showed that higher levels of self-efficacy is linked to better sleep quality. Medical staff with low level of self-efficacy is prone to think that they are not capable of solving emergencies, which may gradually trigger psychological fatigue. Akin and Kas Guner (2019) elucidated that self-efficacy is related to fatigue, and people with low self-efficacy may not be able to effectively cope with its symptoms.

Resilience refers to the ability, result or dynamic process of an individual to successfully adapt to adversity, trauma or other major stressors in the face of adversity (Thapa et al., 2021). Past studies have shown that anxiety or depression in patients with chronic diseases is negatively correlated with resilience, which is a predictor of anxiety and depression (Pang et al., 2021). Awano et al. (2020) found that during the COVID-19 pandemic, medical staff with higher CD-RISC 10 scores were less likely to develop severe depressive symptoms or signs of anxiety. Furthermore, several studies showed that resilience also had a negative predictive effect on insomnia and fatigue. Labrague et al. (2021) pointed out that the level of resilience is closely related to sleep quality, which means that people with higher resilience tend to have higher sleep quality, fewer awakenings, solid sleep depth, and less insomnia. Similarly, Qiu et al. (2020) verified that individuals with higher resilience show higher adaptability to the environment, so they are less vulnerable to fatigue symptoms. It should be emphasized that although resilience and the self-efficacy mentioned above are highly correlated in theory, they play different roles in actual stressful environments. Self-efficacy is related to the pre-belief of nurses that they “can” cope with stress, while resilience is reflected in the actual process of “how” to persist and recover when facing setbacks (Pérez-Jiménez et al., 2024). Therefore, analyzing these two variables as distinct predictors, respectively, is helpful to more accurately reveal their independent mechanism of action in improving mental health status of hemodialysis nurses.

Insomnia and fatigue are also closely related to the mental health status of nurses, especially for individuals with poor sleep quality, anxiety, depression, or other negative emotions (Okun et al., 2018). Many medical staff are prone to insomnia after work, which may be linked to cases of depression and anxiety. This condition seriously threatens the physical and mental health, leading to decreased work efficiency and increased medical errors (Garrouste-Orgeas et al., 2015; Naji et al., 2021). Furthermore, long-term work overload, irregular sleep, poor sleep quality, and lack of understanding and support in the work environment make them vulnerable to physical and mental failure syndrome (Xiao et al., 2020) and chronic fatigue, which has negative effects on their mental health (Ross et al., 2021). High levels of chronic fatigue degree are associated with lower levels of mental health. Meanwhile, perception of sleep plays an important role in predicting reports of daytime fatigue. Insomnia at night causes too short sleep time, which will aggravate the fatigue during the daytime (Harris et al., 2021).

**FIGURE 1:**
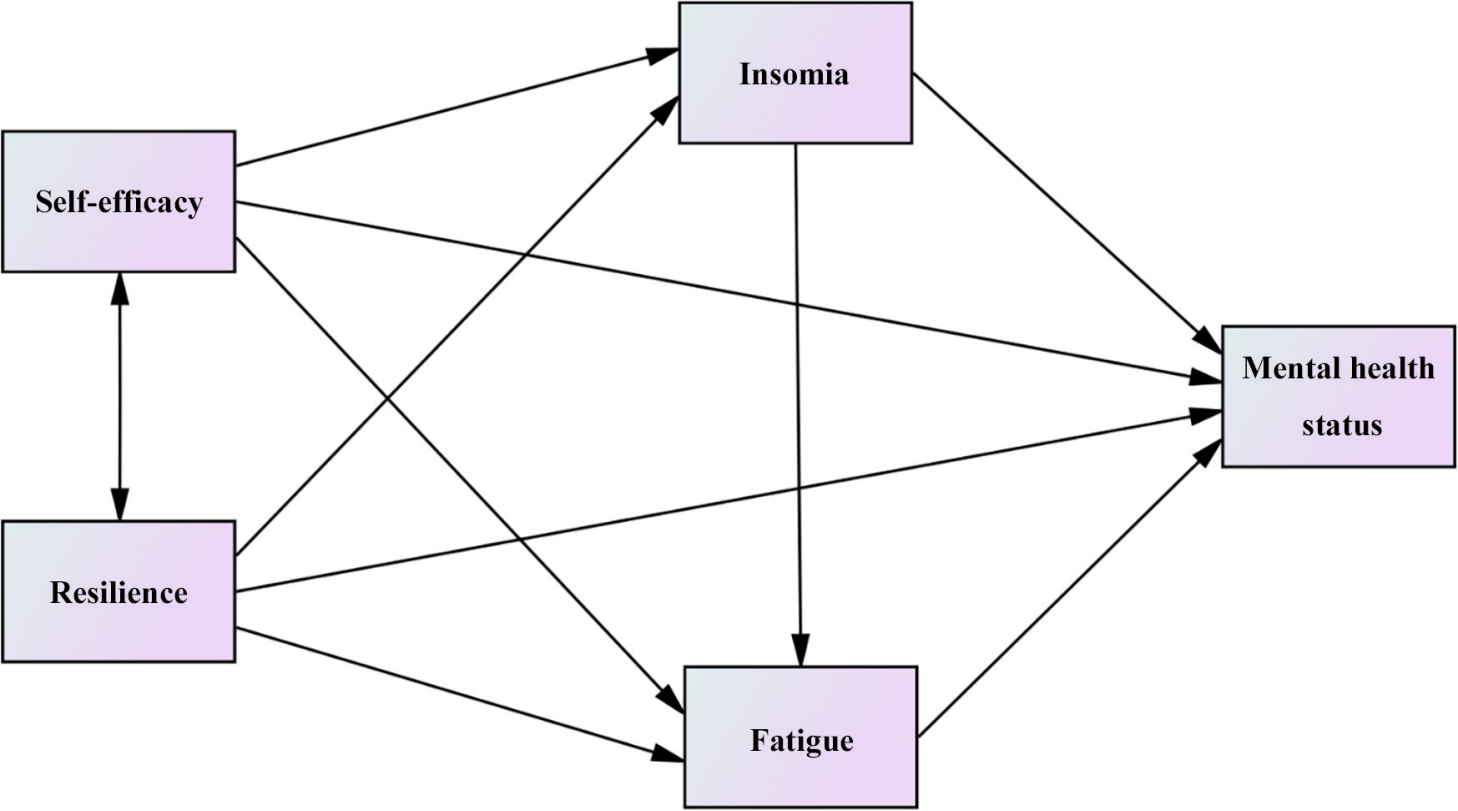
Conceptual framework of hypothesized mediation effects.

Based on the above-mentioned findings of related literature, we propose the following hypotheses and conceptual frameworks (Figure 1):

Hypothesis 1: Self-efficacy/Resilience can directly affect the mental health status.

Hypothesis 2: Self-efficacy/Resilience can affect the mental health status through insomnia.

Hypothesis 3: Self-efficacy/Resilience can affect the mental health status through fatigue.

Hypothesis 4: Self-efficacy/Resilience can affect the mental health status through the chain mediator of insomnia and fatigue.

From the perspective of research design, previous studies mostly discussed the simple correlation and regression between two variables, but did not discuss the interaction among multiple variables and the possible causal order. In addition, when it comes to statistical methods commonly applied in this field, many scholars used *T*-test, one-way ANOVA and multiple regression to analyze their data. Compared with these methods, structural equation model (SEM) can: (1) analyze the relationship among multiple variables simultaneously; (2) estimate latent variables that cannot be directly observed by model analysis of observed variables; (3) test mediating effects and moderating effects and provide quantitative estimates of these indirect effects; (4) provide the significance of path coefficients and offer a series of overall fitting indices (e.g., χ^2^/df, CFI, RMSEA, SRMR, etc.) to determine the fitting effect between theoretical models and actual data (Alzoraiki et al., 2023; Stein et al., 2017). Therefore, we chose SEM to incorporate abstract concepts as latent variables into the model, and to reveal how these core driving variables that cannot be directly observed affect other variables. Meanwhile, we utilized SEM to construct a systematic theoretical model that encompassed both measurement and structural relationships, and to examine the complex causal mechanisms among variables in a more rigorous manner. This methodology is also known as causal model (Bentler, 1980).

Our study aims to explore the relationship between self-efficacy/resilience and the mental health status of hemodialysis nurses, and explore mediating roles of insomnia and fatigue in this context to better understand the their mental health status.

## 2 Materials and methods

### 2.1 Design and participants

A cross-sectional quantitative study was conducted in hemodialysis rooms/centers in Guangdong Province from February 2020 to March 2020, including 1630 hemodialysis nurses aged between 18 and 59. Inclusion criteria: Registered nurses performing first-line clinical care in hemodialysis rooms/centers. Exclusion criteria: (1) Nurses who had not obtained the practice qualification, nurses from other departments, probationer nurses and nurses who took maternity leave, sick leave and personal leave during the survey; (2) Individuals who did not fill in the required items of the questionnaire. This study was approved by the Ethics Committee of the First Affiliated Hospital of Sun Yat-sen University (protocol code: 2022-020). Our research design did not impose any risks to the subjects involved, and informed consent was obtained prior to the analysis in accordance with the Declaration of Helsinki.

### 2.2 Data collection

Our study was conducted by means of an electronic questionnaire. From February to March 2020, an electronic questionnaire was delivered to hemodialysis nurses of various hospitals in Guangdong Province, China. A research group was established, which was responsible for the distribution and collection of questionnaires. After obtaining the approval of the ethics committee of the hospital, the head nurses in charge of each hospital were contacted to get familiar with the purpose and procedure of the survey. The researchers conducted uniform training for the investigators, including the composition and filling requirements of the questionnaire. Finally, the research group checked the questionnaires and eliminated invalid questionnaires. Our exclusion criteria of invalid questionnaires included: inconsistent answer logic; answer time less than 300 s; simple, repeated and abnormal answers; informed disagreement. All questionnaires were found to be valid, with a recovery rate of 100%.

### 2.3 Measurements

#### 2.3.1 Demographic characteristics

The demographic characteristics of participants in this study included gender, age, education level, marital status, title and hospital level.

#### 2.3.2 General self-efficacy scale (GSES)

This study used General self-efficacy scale (GSES). The Chinese version of GSES was used to measure the self-efficacy of the medical staff. The scale was made up of 10 items, with each item scoring between 1 and 4 on an overall scale of 10–40. A higher score indicated a higher level of self-efficacy. The Cronbach’s α of internal consistency using GSES was 0.83 (Lickiewicz et al., 2021).

#### 2.3.3 Connor-Davidson resilience scale (CD-RISC)

To measure nurses’ resilience, we used the Chinese version of Connor-Davidson resilience scale (CD-RISC) that included 25 items related to the ability to face adversity (Wang et al., 2010), with each item scored on a scale of 0–4. This tool had shown sufficient psychometric characteristics when applied to the general population, with Cronbach’s α of internal consistency being 0.89 (Connor and Davidson, 2003).

#### 2.3.4 Insomnia severity index (ISI)

The structure was assessed using Insomnia severity index (ISI), an instrument containing 7 items. Participants were asked to rate each item using a five-point Likert Scale, with an overall score ranging from 0 to 28. In this case, higher scores represented worse levels of insomnia. Among them, 0–7 represented no insomnia, 8–14 represented mild insomnia, 15–21 represented moderate insomnia, and 22–28 represented severe insomnia. The Cronbach’s α of internal consistency of the scale was above 0.80 (Yazdi et al., 2012).

#### 2.3.5 Fatigue scale-14 (FS-14)

This study used 14 items from Fatigue scale-14 (FS-14), including physical fatigue (items 1–8) and mental fatigue (items 9–14). The overall scores ranged from 0 to 14. Fatigue occured when the score was 7 or higher. Cronbach’s α of the two dimensions of the scale were 0.7449 and 0.7953, respectively, and the total Cronbach’s α was 0.7725 (Morriss et al., 1998).

#### 2.3.6 Kessler 10 scale

Kessler 10 Scale was a self-reported measure to assess the presence of mental disorders such as anxiety and depression in the past 4 weeks. The scale contained 10 items, with scores ranging from 1 to 5 and an overall score from 10 to 50. Here, 20 or below represented good mental health status, 20 to 24 represented moderate mental health status, and 25 and above represented poor mental health status. The Cronbach’s α of internal consistency of the scale was 0.88 (Berthelot et al., 2020).

### 2.4 Reliability and validity

All included tools were chosen for their high internal consistency. The Cronbach’s α coefficients of GSES, CD-RISC, ISI, FS-10, and Kessler 10 were 0.920, 0.730, 0.951, 0.905 and 0.950, and the KMO test coefficients were 0.913, 0.905, 0.955, 0.931 and 0.974, respectively. The Bartlett sphericity test also showed statistical significance (*P* < 0.001). Combined with the factor analysis results, the reliability and validity of the questionnaires were considered satisfactory.

### 2.5 Statistical analysis

The means and standard derivations (SD) were calculated to describe the continuous variables, and frequencies with percentages were calculated to describe the dichotomous or categorical variables. Student’ *t*-tests or one-way ANOVA were used for difference analyses and Spearman correlation analysis was used to examine the correlation between the two variables. Multiple comparisons were conducted using the Bonferroni test.

Furthermore, Maximum Likelihood Estimation (MLR) was used to estimate the model. The absolute goodness of fit indices included GFI, AGFI, SRMR, RMSEA, and χ^2^/df; the relative goodness of fit indices included CFI, TLI, NFI, and IFI. In our study, all these indices were greater than 0.90. SRMR and RMSEA were all less than 0.05 and χ^2^/df was less than 5, indicating a good model fitting effect (Hu and Bentler, 1999). Additionally, the Bootstrap method was used to analyze the mediation effect and the number of repeated samples was set to 5000. When the 95% CI did not contain 0, the mediation effect was considered to be significant. If 0 was included, it was labeled as not significant.

*P*-values were two-sided with alpha set at <0.05. The statistical analysis was performed with SPSS (version 26.0) and AMOS (versions 24.0).

## 3 Results

### 3.1 Comparison of characteristic data and mental health status scores of hemodialysis nurses

A total of 1630 hemodialysis nurses with an average age of 33.20 (18–59) years, participated in the study. More than half of the hemodialysis nurses received a bachelor’s degree or above (52.03%) and most of them were married (70.49%) and working in tertiary hospitals (72.33%). Significant differences were observed in term of education level and hospital level. The results of multiple comparisons showed that hemodialysis nurses with a bachelor’s degree or above in education had a higher Kessler 10 score than those with high/vocational school (*P* = 0.003) or junior college (*P* = 0.017) education. In terms of the hospital level, hemodialysis nurses working in tertiary hospitals had a higher Kessler 10 score than those working in primary (*P* = 0.004) and secondary (*P* < 0.001) hospitals. More details are presented in Table 1.

**TABLE 1:** Comparison of scores on general characteristics and mental health status in hemodialysis nurses.

Variable	*n* (%)	Kessler 10 scale ($\bar{\text{x}}\pm \text{s}$)	Statistical value	*P-*value
Total	1630	20.16 ± 7.47		
Gender			−0.034^a^	0.973
Female	1526 (93.62)	20.15 ± 7.40		
Male	104 (6.38)	20.18 ± 8.48		
Age			1.871^b^	0.132
≤30	744 (45.65)	19.78 ± 7.81		
31–40	555 (34.05)	20.68 ± 7.19		
41–50	299 (18.34)	19.99 ± 7.05		
>50	32 (1.96)	21.38 ± 7.68		
Educational level			7.444^b^	0.001
High/vocational school	130 (7.97)	18.47 ± 7.08		
Junior college	652 (40.00)	19.70 ± 7.60		
≥College	848 (52.03)	20.77 ± 7.37		
Marital status			0.065^b^	0.937
Unmarried	449 (27.55)	20.07 ± 7.69		
Married	1149 (70.49)	20.20 ± 7.37		
Other*	32 (1.96)	19.91 ± 7.96		
Title			1.556^b^	0.198
Nurse	479 (29.39)	19.68 ± 7.67		
Nurse practitioner	566 (34.72)	20.11 ± 7.63		
Nurse-in-charge	486 (29.82)	20.45 ± 7.09		
Vice-senior or senior	99 (6.07)	21.20 ± 7.27		
Hospital level			16.756^b^	<0.001
Primary	67 (4.11)	17.76 ± 7.11		
Secondary	327 (20.06)	17.91 ± 6.25		
Tertiary	1179 (72.33)	20.92 ± 7.61		
Other	57 (3.50)	20.05 ± 8.31		

*Including, divorced, remarried, and spouse loss; ^a^student’ *t*-tests; ^b^one-way ANOVA.

### 3.2 Correlation of GSES, CD-RISC, ISI, FS-14, and Kessler 10 in hemodialysis nurses

The scores of GSES and CD-RISC were (6.76 ± 1.11) and (58.36 ± 15.74), respectively. ISI and FS-14 scores were (5.62 ± 4.65) and (6.51 ± 3.04), respectively. We also identified that 443 hemodialysis nurses (27.18%) had mild insomnia, 63 (3.86%) had moderate insomnia, 12 (0.74%) had severe insomnia, and 741 (45.46%) had fatigue. The score of Kessler 10 was (20.16 ± 7.47), and 605 (37.12%) individuals fit the conditions of poor mental health status. According to Table 2, the correlation analysis of scales showed that GSES/CD-RISC was negatively correlated with ISI, FS-14, and Kessler 10 (*P* < 0.001), respectively. Except for the two dimensions of the FS-14 scale, the other dimensions were also significantly correlated, as shown in Table 3.

**TABLE 2:** Correlation analysis among scales.

Scale	GSES	CD-RISC	ISI	FS-14	Kessler 10
GSES	1.000				
CD-RISC	0.676***	1.000			
ISI	−0.310***	−0.288***	1.000		
FS-14	−0.334***	−0.313***	0.513***	1.000	
Kessler 10	−0.455***	−0.476***	0.573***	0.635***	1.000

***P < 0.001; GSES, General self-efficacy scale; CD-RISC, Connor-Davidson resilience scale; ISI, Insomnia severity index; FS-14, Fatigue scale-14.

**TABLE 3:** Correlation analysis of scores for variables.

Scale and dimension		1	2	FS-14		CD-RISC					Kessler 10	
				3	4	5	6	7	8	9	10	11
1. ISI		1.000										
2. GSES		−0.310***	1.000									
FS-14	3. Physical	0.563***	−0.369***	1.000								
	4. Mental	0.026	−0.013	0.133***	1.000							
CD-RISC	5. Spiritual influence	−0.129***	0.313***	−0.113	0.009	1.000						
	6. Control	−0.221***	0.560***	−0.266	0.019	0.358***	1.000					
	7. Acceptance	−0.296***	0.635***	−0.372	−0.012	0.466***	0.674***	1.000				
	8. Tolerance	−0.258***	0.639***	−0.314	0.004	0.396***	0.742***	0.747***	1.000			
	9. Competence	−0.266***	0.607***	−0.335***	0.004	0.385***	0.748***	0.733***	0.811***	1.000		
Kessler 10	10. Depression	0.572***	−0.408***	0.664***	0.085***	−0.163***	0.333***	−0.453***	−0.356***	−0.408***	1.000	
	11. Anxiety	0.548***	−0.465***	0.663***	0.073***	−0.177***	−0.388***	−0.508***	−0.414***	−0.471***	0.895***	1.000

***P < 0.001; GSES, General self-efficacy scale; CD-RISC, Connor-Davidson resilience scale; ISI, Insomnia severity index; FS-14, Fatigue scale-14 scale.

### 3.3 The fitting effect of structural equation model and path analysis

**FIGURE 2:**
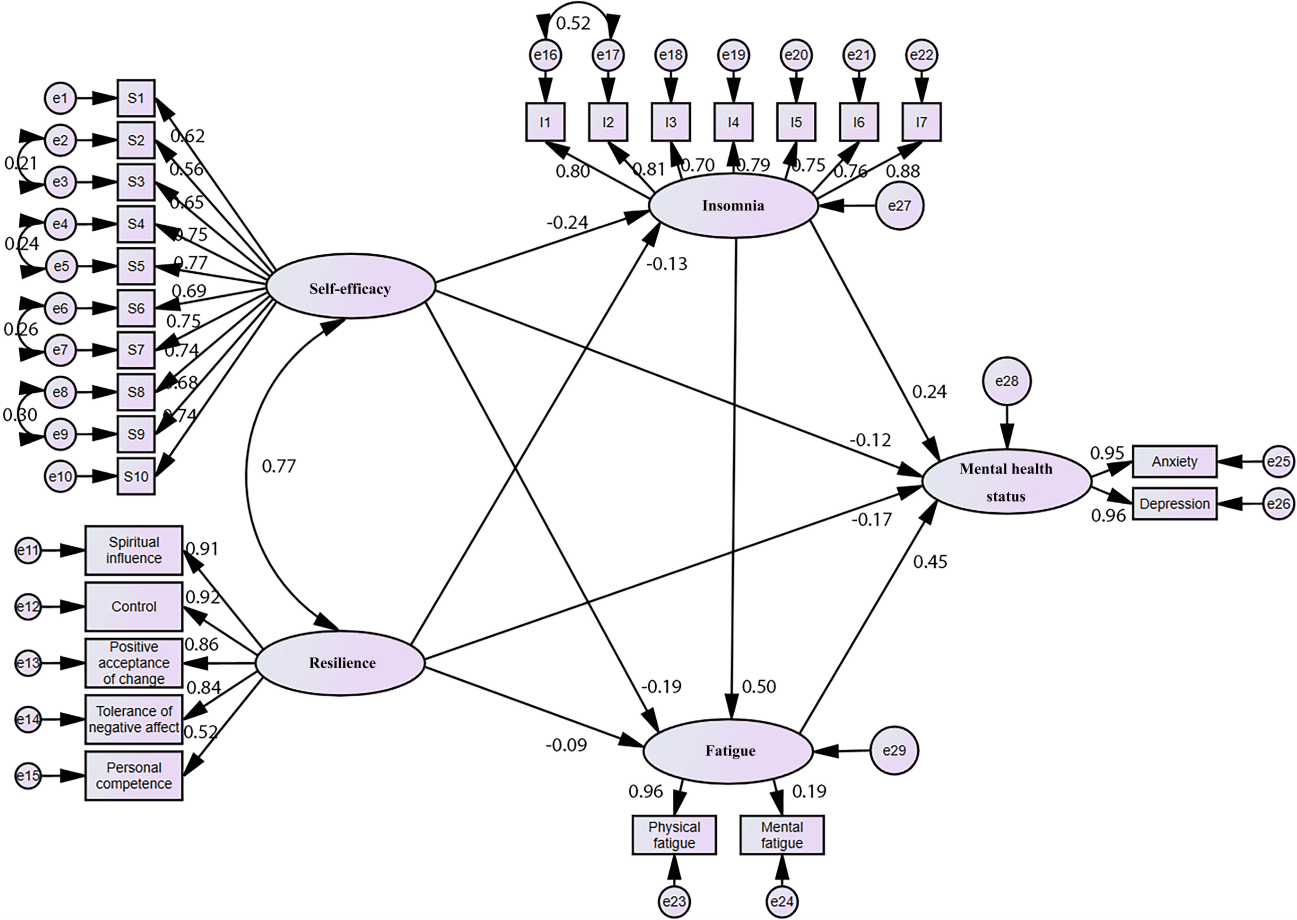
Final structural equation model with standardized path coefficients.

We had analyzed a large body of literature to outline our hypotheses and conceptual frameworks and propose a SEM to quantify the relationship between self-efficacy, resilience, insomnia, fatigue, and mental health status. The graphical form of our final model is represented in Figure 2. We considered self-efficacy and resilience were taken as independent variables, insomnia and fatigue as mediators, and mental health status as dependent variables.

In the proposed model, GSES and ISI were one-dimensional and CD-RISC had five dimensions, i.e., spiritual influence, control, acceptance of change, tolerance of negative feelings, and competence. FS-14 included physical fatigue and mental fatigue, and Kessler 10 included anxiety and depression. After model modification, the final model fitting results were χ^2^ = 1315.092, df = 284, χ^2^/df = 4.631, *P* < 0.001, GFI = 0.940, AGFI = 0.926, SRMR = 0.0341, RMSEA = 0.047, 95%CI = (0.045,0.050), CFI = 0.964, TLI = 0.959, NFI = 0.955, IFI = 0.964, indicating a satisfactory fitting effect.

Structural equation model analysis results and path coefficients of each pathway are shown in Table 4. As shown, the path coefficients of each pathway had statistical significance (*P* < 0.05), indicating that all of them were valid. Insomnia and fatigue played multiple mediating roles in the relationship between self-efficacy/resilience and mental health status. Our results also pointed out that self-efficacy significantly negatively predicted the degree of insomnia (β = −0.236), fatigue (β = −0.186) and mental health status (β = −0.123). Resilience also significantly negatively affected the degree of insomnia (β = −0.131), fatigue (β = −0.086) and mental health status (β = −0.172).

**TABLE 4:** Path analysis.

Path	β	95%CI
Self-efficacy → insomnia	−0.236	(−0.334, −0.137)***
Self-efficacy → fatigue	−0.186	(−0.278, −0.110)***
Self-efficacy → mental health status	−0.123	(−0.207, −0.028)*
Resilience→ insomnia	−0.131	(−0.227, −0.036)**
Resilience→ fatigue	−0.086	(−0.159, −0.012)*
Resilience→ mental health status	−0.172	(−0.241, −0.106)***
Insomnia → mental health status	0.240	(0.096, 0.353)**
Fatigue → mental health status	0.451	(0.295, 0.659)***
Insomnia → fatigue	0.499	(0.417, 0.578)***

*P < 0.05, **P < 0.01, ***P < 0.001.

Subsequently, it was identified that the effect of resilience on mental health status was greater than that of self-efficacy. In addition, the effects of self-efficacy and resilience on the degree of insomnia were greater than the effects of fatigue, and the effects of self-efficacy were greater than the effects of resilience. The degree of insomnia and fatigue had a significant positive effect on mental health status (β = 0.240 and 0.451), and the effect of fatigue was greater than that of insomnia. In addition, insomnia also significantly affected fatigue status (β = 0.499). Among all the factors that affect mental health status, the influence of fatigue was the biggest.

### 3.4 Multiple mediation effects of insomnia and fatigue between self-efficacy/resilience and the mental health status of hemodialysis nurses

The Bootstrap program of AMOS was used to repeat the sampling for 5000 times, and the 95%CI was taken to test the significance of the mediation effect. According to Table 5, 95%CI corresponding to each mediating pathway did not contain 0, indicating that each indirect effect pathway was significant, and there were multiple mediation effects between self-efficacy/resilience and the mental health status of hemodialysis nurses.

**TABLE 5:** Analysis of multiple mediation effects of insomnia and fatigue in hemodialysis nurses.

	β	95%CI	Effect size/%
Direct effect 1: self-efficacy → mental health status	−0.123	(−0.207, −0.028)*	38.80
Indirect effect 1: Self-efficacy → insomnia → mental health status	−0.057	(−0.101, −0.023)**	17.98
Indirect effect 2: Self-efficacy → fatigue → mental health status	−0.084	(−0.163, −0.038)***	26.50
Indirect effect 3: Self-efficacy → insomnia → fatigue → mental health status	−0.053	(−0.100, −0.025)***	16.72
Total indirect effect 1	−0.194	(−0.284, −0.121)***	61.20
Total effect 1	−0.317	(−0.408, −0.221)***	100.00
**Comparison of mediating effects:**			
ID1 and ID2	0.027	(−0.047, 0.134)	-
ID1 and ID3	−0.004	(−0.055, 0.064)	-
ID2 and ID3	−0.031	(−0.093, 0.007)	-
Direct effect 2: resilience → mental health status	−0.172	(−0.241, −0.106)***	63.47
Indirect effect 4: resilience → insomnia → mental health status	−0.031	(−0.069, −0.008)**	11.44
Indirect effect 5: resilience → fatigue → mental health status	−0.039	(−0.088, −0.007)*	14.39
Indirect effect 6: resilience → insomnia → fatigue → mental health status	−0.029	(−0.068, −0.009) **	10.70
Total indirect effect 2	−0.099	(−0.162, −0.041)***	36.53
Total effect 2	−0.271	(−0.365, −0.181)***	100.00
**Comparison of mediating effects:**			
ID4 and ID5	0.007	(−0.041, 0.066)	-
ID4 and ID6	−0.002	(−0.036, 0.035)	-
ID5 and ID6	−0.009	(−0.052, 0.034)	-

*P < 0.05, **P < 0.01, ***P < 0.001

With self-efficacy as the independent variable and insomnia and fatigue as the chain mediating pathway, the corresponding 95%CI was (−0.100, −0.025), and the effect value was −0.053. The 95%CI corresponding to the mediating chain pathway with resilience as the independent variable was (−0.068, −0.009), and the effect value was −0.029. In this case, 95%CI also did not contain 0, and the chain mediation effect was significant. Meanwhile, the 95%CI of direct effect with self-efficacy as an independent variable was (−0.207, −0.028), and the effect value was −0.123. The 95%CI of direct effect with resilience as an independent variable was (−0.241, −0.106), and the effect value was −0.172. The 95%CI corresponding to the two direct effect pathways also did not contain 0, indicating that insomnia and fatigue played a partial mediating role in the relationship between self-efficacy/resilience and mental health status.

The comparison results of all indirect effect pathways showed that the 95%CI of the difference between the effect values of all indirect effect pathways contained 0, indicating that there was no difference in their overall effects. Finally, the contribution of the mediation effect was measured by calculating the effect size of each pathway by dividing the standardization effect of each pathway by the total standardization effect. Among them, with self-efficacy as an independent variable, the total mediation effect size was 61.20%, larger than the effect size of the direct effect. However, with resilience as an independent variable, the total mediation effect size was 36.53%, which was less than the effect size of the direct effect.

## 4 Discussion

In recent years, with the increasing number of ESRD patients, the demand for hemodialysis has been constantly surging. In addition, under the continuous influence of COVID-19 pandemic, hemodialysis nurses are prone to have anxiety, depression, and other negative emotions due to the challenges imposed by the pandemic, heavy workload, and a large number of hemodialysis patients (Sriharan et al., 2021; Walton et al., 2020). In our study, the proportion of self-reported poor mental health status was 37.12%, indicating their poor mental condition. This condition did not only affect the mental health of hemodialysis nurses, but also the quality of care for patients, potentially leading to major medical errors (Garrouste-Orgeas et al., 2015). We also found that the mental health status of hemodialysis nurses varied significantly by educational and hospital level. Hemodialysis nurses with high educational degree contributed to mental health problems, which accorded with previous findings (Jiang et al., 2022; Wang et al., 2021). Highly educated nurses usually took on more positions (e.g., quality control and management work) and cared for more critically ill patients. In addition, hemodialysis nurses working in tertiary hospitals had a poorer mental health status than those working in primary and secondary hospitals. This might be due to the fact that tertiary hospitals had a large workload in prevention and control of COVID-19 pandemic and received more critically ill patients (Yun et al., 2024).

According to the characteristics of hemodialysis work, hemodialysis nurses are faced with the following pressure sources: (1) Special working environment: Hemodialysis room machines produce noise, radiation, and other unsafe factors (Naji et al., 2022; Saleem et al., 2021); the smell of the patient’s feces can cause discomfort. (2) Heavy workload: Due to sudden changes in the patient’s condition, nurses often work overtime (Watanabe et al., 2021; Yıldırım and Şentürk, 2023); unreasonable scheduling in some hospitals can affect the rest of nurses. (3) Occupational infection: HBV and HCV infection are the most common infections in hemodialysis patients (Bernieh, 2015), so hemodialysis nurses become the high-risk group for these virus infections; under the influence of COVID-19 pandemic, nurses may risk cross-infection due to close contact with patients during treatment (Yıldırım and Şentürk, 2023). (4) Social prejudice: Role positioning of nursing work is unclear, lack of understanding and social support, and labor value is not recognized and respected (Dziedzic et al., 2025). (5) Update of professional technology: With the continuous development of medical technology, hemodialysis equipment is constantly updated (El et al., 2022), which requires nurses to also constantly update their knowledge and master the use of various new equipment.

In the past, researchers have mainly explored relationships between self-efficacy, resilience, insomnia, fatigue, and mental health status, but few articles have explored the functional relationship and mediating pathway among these variables. Therefore, based on previous research results, this study used SEM to explore relationships among these variables and multiple mediation effects. Theoretically, this model reveals that improving the self-efficacy and resilience of hemodialysis nurses can reduce the risk of anxiety and depression. The findings of this study indicated that each pathway was significant, which meant that all the four hypotheses proposed at the beginning of the study were successfully verified.

Furthermore, our study found that self-efficacy can negatively impact the mental health status of hemodialysis nurses and was a predictor of mental health, which was consistent with the results presented by other scholars (Azemi et al., 2022; Maciejewski et al., 2000). Our results also pointed out that people with lower self-efficacy were more vulnerable to anxiety and depression. The effect of self-efficacy on nurses’ mental health status can also be generated through three mediating pathways: the mediation effect of insomnia, the mediation effect of fatigue and the chain mediation effect of insomnia and fatigue.

It was also identified that self-efficacy can negatively predict the severity of insomnia. Nurses with high self-efficacy are not easily disturbed by various stressors during sleep, so as to maintain the stability of sleep and circadian rhythm (Schmitt et al., 2017). According to the 3P-disease model (Wright et al., 2019), low self-efficacy is a predisposing factor for insomnia. Moreover, this study found that self-efficacy can also predict the state of fatigue, which was consistent with Hou et al.’s (2020) conclusions. Furthermore, individuals with high levels of self-efficacy usually have self-confidence. They believe that they are competent enough to finish complicated tasks and have more courage to face setbacks (Wachs et al., 2018), which plays a positive role in reducing individual fatigue.

The degree of insomnia and fatigue also had a significantly negative impact on the mental health status of nurses. For people with better sleep quality, their mental health level is usually higher, a conclusion that is consistent with the results of other studies (Pappa et al., 2020; Scott et al., 2021). Long-term sleep-deprivation and light sleep will cause memory decline, inattention, and other psychological problems (Zare et al., 2019). Some contemporary studies conducted in China already demonstrated that nurses are a high-risk group for fatigue syndrome (Li et al., 2020) and that chronic fatigue can increase the anxiety and depression levels of nurses, which in turn can increase the experience of negative emotions and fatigue, leading to a vicious cycle (Zhan et al., 2020). In addition, long-term insomnia can aggravate fatigue, because both body and mind cannot get sufficient rest.

Our study showed that the total indirect effect of self-efficacy on the mental health status of hemodialysis nurses was greater than the direct effect, indicating that the influence degree of self-efficacy on mental health status mainly depended on the severity of insomnia and fatigue. Therefore, while improving the level of self-efficacy of nurses, we should pay more attention to improving the quality of sleep and relieving fatigue to effectively upgrade their mental health status and mental health. It can be used as a critical point of psychological intervention for nurses. For example, mindfulness therapy has been widely used to treat and relieve anxiety, depression and other psychological problems (Kwok et al., 2019), which can improve self-efficacy, have a positive impact on fatigue (McConville et al., 2017), and then maintain the mental health of nurses. Cognitive-behavioral therapy can also improve self-efficacy or directly relieve fatigue-related symptoms (Turner et al., 2016). Therefore, hospitals should design a mindfulness-based stress reduction plan based on the characteristics of occupational stress of hemodialysis nurses during the epidemic. Professional psychotherapists organize nurses to participate in systematic training and introduce the relevant knowledge and skills of mindfulness-based stress reduction. Meanwhile, these professionals lead nurses to participate in various forms of stress reduction training during shift handovers or breaks, including mindfulness breathing, mindful sleep and eating, and mindfulness meditation, and instruct them to integrate each training with their daily lives (Kwok et al., 2019; Yıldırım and Çiriş, 2022). Each training lasts for 1–1.5 h, for 8 weeks. After each training, professionals assign corresponding practice tasks and require nurses to conduct at least 15–20 min of mindfulness practice every day and record their feelings and insights from the practice (Liu et al., 2023).

Additionally, our study also found that resilience can negatively predict the mental health status, which was generally regarded as an adaptive protection mechanism for mental diseases, helping individuals adapt to the environment and regain strength after facing setbacks (Ristevska-Dimitrovska et al., 2015). Individuals with a high level of resilience usually are less prone to face episodes of anxiety and depression, which is consistent with previous studies (Awano et al., 2020; Mirzaei et al., 2022).

Resilience can also indirectly affect the mental health status of hemodialysis nurses through three mediating pathways. Firstly, it has a negatively predictive effect on insomnia, which means that higher levels of resilience make individuals less prone to insomnia. Similarly, Cheng et al. (2020) have previously claimed that resilience is a good psychological adaptability when facing pressure and frustration. When nurses are stimulated by various stressful factors, it might get challenging to adjust and adapt to the low level of resilience, resulting in poor sleep quality and frequent awakening (Seelig et al., 2016). Therefore, resilience is an important protective factor against insomnia in medical staff. Secondly, resilience also has a negatively predictive effect on fatigue. Nurses with higher resilience may show stronger pressure resistance when they complete difficult puncture work and emergency dialysis (Labrague and de Los, 2021), so they are less susceptible to fatigue symptoms at work. In order to safeguard the mental health of hemodialysis nurses during the pandemic, hospital administrators should implement proactive measures to sustain resilience in hemodialysis nurses.

Our model also found that the direct effect of resilience on nurses’ mental health status was greater than the total indirect effect, suggesting that improving the level of resilience can also improve mental health status. Thus, targeted resilience training can be carried out. Effective resilience training starts from cognition, emotion, spirit, and behavior, and can enhance individuals’ emotional management ability, problem-solving skills, and self-awareness (Magtibay et al., 2017). Evidently, improving insomnia and fatigue at the same time can be more effective in maintaining mental health, improving work productivity, and reducing medical errors.

However, we also need to point out that, due to the lack of targeted longitudinal studies or experimental studies, the directions of the pathways in our model are based on theoretical assumptions and that alternative causal relationships (e.g., poor mental health status leading to lower self-efficacy/resilience) cannot be ruled out. Therefore, more studies are recommended to establish causality and test effectiveness of resilience/self-efficacy training in the future.

## 5 Limitation

The findings of this study need to be interpreted in consideration of the following limitations. First, all participants were recruited from Guangdong province, which might cause selection bias and limit the generalizability of our findings. As an economically developed province in China, Guangdong Province may have differences in the level of medical resources, hospital management system, work pace and cultural environment compared with inland China and other countries. These specific regional factors may affect the types and levels of stress perceived by nurses, which in turn may limit the generalizability of the conclusions of our study in other provinces or countries. Future studies are needed to further verify the applicability of the model in this study through cross-regional and multi-center approaches. Second, questionnaire data were all self-reported, which can lead to social desirability bias, as well as underestimation or overestimation of our reported results. Third, cross-sectional studies limit any conclusions on the causal relationship between self-efficacy/resilience and the mental health status of hemodialysis nurses. Therefore, longitudinal or experimental studies are needed to make any causal claims in conjunction with the associations among the variables. Fourth, this data was collected from February to March 2020 in Guangdong, which was the epicenter of the initial COVID-19 pandemic. Compared with non-pandemic periods, hemodialysis nurses, in addition to completing basic hemodialysis care work, also needed to fulfill their responsibilities in pandemic prevention and control and be on standby at any time for the pandemic. This would inevitably extend working hours, disrupt the work-life balance, and also pose a risk of infection. Therefore, this unique high-stress environment, as an important confounding factor, might have amplified the mediating effects of insomnia and fatigue. Therefore, during non-pandemic periods, we can conduct similar research in accordance with the theoretical framework of this study to observe whether the results are reproducible. If there are differences in the results of two surveys, we should analyze the reasons for the differences.

## 6 Conclusion

Our study found that the specific demands of hemodialysis nursing bring various stressors that can negatively affect the mental health status of hemodialysis nurses, which makes them vulnerable to anxiety, depression, and other negative emotions. The results of multiple mediation effects analysis based on SEM suggested that self-efficacy and resilience can directly affect the mental health status of these nurses, and also indirectly affect their mental health status through insomnia and fatigue. In other words, these results showed that insomnia and fatigue can also directly affect the mental health of these individuals. Therefore, it is necessary to think of effective means to improve the self-efficacy and resilience of hemodialysis nurses inserted in this context. Hospitals should prioritize self-efficacy/resilience-building programs and fatigue management interventions. Each hemodialysis room should have a reasonable schedule to reduce the severity of insomnia and fatigue to guarantee that nurses have more quality of life at work, which can significantly impact their journeys toward better mental health.

## Data Availability

The raw data supporting the conclusions of this article will be made available by the authors, without undue reservation.
